# An undergraduate medical curriculum framework for providing care to transgender and gender diverse patients: A modified Delphi study

**DOI:** 10.1007/s40037-021-00692-7

**Published:** 2021-11-18

**Authors:** Rachel H. Ellaway, Nicole L. Thompson, Claire Temple-Oberle, Danièle Pacaud, Helena Frecker, Theodore J. Jablonski, James Demers, Fiona Mattatall, Joe Raiche, Andrea Hull, Rabiya Jalil

**Affiliations:** 1 Department of Community Health Sciences, Cumming School of Medicine, University of Calgary, Calgary, Alberta Canada; 2 Department of Obstetrics and Gynecology, University of British Columba, Vancouver, British Columbia Canada; 3 Department of Plastic Surgery, Cumming School of Medicine, University of Calgary, Calgary, Alberta Canada; 4 Department of Pediatric Endocrinology, Cumming School of Medicine, University of Calgary, Calgary, Alberta Canada; 5 Department of Obstetrics and Gynecology, University of Toronto School of Medicine, Toronto, Ontario Canada; 6 Department of Family Medicine, Cumming School of Medicine, University of Calgary, Calgary, Alberta Canada; 7 Queer Education Foundation, Calgary, Alberta Canada; 8 Department of Obstetrics and Gynecology, Cumming School of Medicine, University of Calgary, Calgary, Alberta Canada; 9 Department of Psychiatry, Cumming School of Medicine, University of Calgary, Calgary, Alberta Canada

**Keywords:** Transgender, Gender nonconforming, Curriculum, Syllabus, Safety, Delphi, Canada

## Abstract

**Introduction:**

The lack of attention to transgender and gender diverse (TGD) people in undergraduate medical education (UME) is a point of concern, particularly among medical students. A project was undertaken to develop a UME curriculum framework for teaching the healthcare needs of TGD people.

**Methods:**

Using a modified Delphi methodology, four rounds of surveys were presented to an expert stakeholder group that included content experts, generalist physicians, UME teaching faculty, and medical students. Questions covered what content should be taught, who should teach the content, and how much time should be dedicated for this teaching. Once the Delphi process was complete, feedback on the provisional framework was sought from members of the TGD community to ensure it represented their needs and perspectives.

**Results:**

71 panel members and 56 community members participated in the study. Core values included the scope of the framework, and topics such as inclusivity, and safety in practice and in teaching. The framework included terminology, epidemiology, medical and surgical treatment, mental health, sexual and reproductive health, and routine primary care. There was also guidance on who should teach, time to be allocated, and the learning environment.

**Discussion:**

There is a clear need to train tomorrow’s doctors to provide competent and respectful healthcare services to and for TGD patients. Although local factors will likely shape the way in which this framework will be implemented in different contexts, this paper outlines a core UME-level curriculum framework for Canada and, potentially, for use in other parts of the world.

**Supplementary Information:**

The online version of this article (10.1007/s40037-021-00692-7) contains supplementary material, which is available to authorized users.

## Introduction

Lesbian-Gay-Bisexual-Transgender-Queer (LGBTQ+) people often face significant health disparities, which have been linked to social stigma, discrimination, and failure to respect their civil and human rights [1, 2]. Transgender and gender diverse people (TGD) people in particular tend to experience even greater disparities because of additional issues including bias in both medical training and practice as well as a lack of qualified providers and socioeconomic barriers [3]. These disparities tended to become more acute during the COVID-19 pandemic [4].

The inadequacy of training for health professionals to work with LGBTQ+ patients is a longstanding concern [5–9]. Medical students and TGD patients have been particularly concerned with this issue [10–12]. Despite this, it has been observed that targeted teaching and experiential learning can improve student competence and confidence in working with TGD patients [13–16].

A recent review of the Canadian landscape identified the lack of practitioner knowledge as a key barrier to TGD persons accessing primary care [17]. Obedin-Maliver et al. [5] found that more than a third of North American schools had no core LGBTQ+ content in their curricula, and even those that did tended to focus more on LGB and intersex topics than on those specific to TGD patients. Obedin-Maliver et al. also noted several enabling factors for expanding LGBTQ+ teaching, including access to appropriate curricular material, faculty competent in LGBTQ+-related teaching, and LGBTQ+ content in national examinations and accreditation standards [5]. More recently, Nolan et al. [18] reflecting on the causes of the continued absence of TGD topics in medical education, noted a *“paucity of objective educational intervention outcomes measurements, absence of long-term follow-up data, and varied nature of intervention types” *and that *“a clear best practice for transgender curricular development has not yet been identified in the literature.”* Even where educational interventions have had positive outcomes in building knowledge, issues such as implicit bias remain harder to address [19]. Without a clear and integrated approach to training medical students to provide care to TGD patients, the disproportionate discrimination and barriers to care TGD that individuals experience within the healthcare system will likely continue [20, 21]. Given this inequity and the worrying levels of trauma experienced by TGD individuals in their interactions with the healthcare system [22], training cannot solely be a matter of skills and knowledge, it needs to be trauma-informed to address these longstanding and systemic issues [23].

In 2017, medical students at the University of Calgary undertook a curriculum review of gender diverse content in pre-clerkship training. From this review, a three-hour module was developed and piloted in 2018. Post-session interviews were conducted with medical students, physicians, and TGD community member participants for evaluation and quality improvement purposes. These data generated many questions regarding the content, processes, timing, and assessment of how medical students should be trained to provide care to TGD patients. Using this as a *de facto* needs assessment, we came together as a study team to explore what a core curriculum framework to prepare Canadian medical students to provide TGD affirming care could look like. In this paper, we report on the resulting study, the objective of which was to develop a UME curriculum framework for teaching the healthcare needs of TGD people through a consensus and consultation process with Canadian medical professionals, educators, learners, and TGD community members.

## Methods

### Study design

Because of the significant variation in TGD rights and access to healthcare around the world, we limited the scope of this study to the Canadian context where its Human Rights Act explicitly covers “gender identity and gender expression to the list of prohibited grounds of discrimination” [24]. We focused on undergraduate medical education as it provides the foundational generalist training for all physicians regardless of future practice. This reflected a focus on core training in TGD health rather than the specialty-specific knowledge, skills, and attitudes in residency education.

Given the paucity of attention paid to TGD teaching in health professions education, we could not depend on harvesting the literature or other reports to identify best practices. Rather, we employed a consensus methodology [25], informed by the outline reporting guidelines set out by Humphrey-Murto et al. [26] and Hasson et al. [27]. We note, however, that our approach was more formative and exploratory than the strict reductive convergence approaches used in other consensus studies and as such we were more focused on generating our framework and validating it with members of the TGD community than on validating a pre-existing framework within a modified Delphi process.

**Fig. 1:**
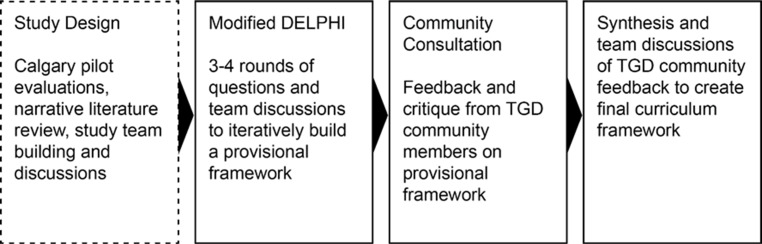
Overall study design and flow

Our overall study design was based around three phases (Fig. 1): A Delphi process to generate a draft curriculum framework; a consultation phase with TGD community members; and a final synthesis phase revising the final curriculum framework through iterative discussion and group writing.

### Delphi phase

We anticipated that it would take four Delphi rounds to cover the different dimensions of a curriculum framework. The first round focused on the TGD objectives while subsequent rounds explored curricular issues such as who should teach TGD material and how much time should be dedicated to teaching TGD concepts. The specific details of each subsequent round were based on the analysis of the results from preceding rounds. The study team kept notes and memos of broader emerging issues and concerns. Initial topics and issues were based on the 2018 pilot UME curriculum developed at the University of Calgary and from a narrative review of the literature (outlined in the introduction section). Given the extent of the negative experiences TGD patients have had in interacting with the healthcare system [1–4, 11], we also took a trauma-informed approach that meant we specifically attended to dimensions of safety, connection, and emotion [23] within the curriculum framework, and to stakeholder communication and involvement around it [28].

Draft instruments for each round were developed by RHE and NLT and reviewed by the study team. The four instruments are provided in Appendix 1 of the Electronic Supplementary Material. An invitation to participate was sent to the panel email list with two follow-up reminders. Each round was closed two weeks after the last reminder was sent. RHE and NLT generated a report from each round, and this was provided to the rest of the team for discussion. An abstracted report of the findings from the previous round was provided to panel members at the start of the next round instrument.

All four Delphi stages and the community consultation phase were conducted online using the Qualtrics survey platform hosted through the University of Calgary (Qualtrics, Provo, UT).

We recruited reviewers from four groups: physicians providing specialist care to TGD persons (surgeons, endocrinologists, psychiatrists etc.); generalist physicians providing primary care to TGD persons (family doctors); UME leaders with perspectives on both the inclusion and exclusion of material from UME curricula; and medical students who had advocated for TGD inclusion in medical education. Physicians were recruited using invitations sent to university departmental email lists and through national physician groups with a TGD patient focus, UME leaders were recruited through invitations sent to local and national email lists, and students were recruited through invitations sent to local and national medical student organizations’ email lists. Participation was anonymous and reflected a convenience sample of volunteers. However, we were able to ensure (from the email addresses) that there were participants from all four groups. Cross-Canada involvement was reflected in having participants from British Columbia, Alberta, Ontario, Quebec, and Nova Scotia. Respecting the confidential and anonymous nature of the study, we did not collect any other panel member demographic data.

#### Analysis and synthesis:

For rating questions we used 5‑option Likert scales and scored them with neutral as zero (i.e, strongly agree (+2), agree (+1), neutral (0), disagree (−1), strongly disagree (−2)). When items were presented for final confirmation, we used a three-item scale (i.e., ‘High priority, definitely include’ (+1); ‘Medium priority, may include’ (0); ‘Low priority—do not include’ (−1)). The aggregate score for each item was the sum of individual scores divided by the total number of responses. Items for each question were then ranked based on this aggregate score. Given the subjective and multifactorial judgments associated with defining a TGD curriculum framework for Canadian UME and our formative and generative focus, we did not define *a priori *convergence/consensus thresholds. Rather, where there was a clear clustering of items around high and low aggregate scores the gap between them was taken as the threshold for inclusion/exclusion. Where there was a unimodal continuum of aggregate item rating scores, the ranked list was presented to the panel in the subsequent round for confirmation. Each rating question was followed by a free-text box for comments and feedback. Additional items suggested in one round were added to the next round instrument, and where issues of terminology or framing were raised, these were discussed and resolved within the study team.

RHE and NLT generated reports on the results of each round which were circulated to the study team for reflection and comment. Points for clarification or disagreement were discussed to reach in-team consensus on how the results should be interpreted. From this, RHE and NLT developed a draft of the instrument for the next round. This was circulated within the team and again, through discussion, a final version was confirmed and distributed. At the end of the fourth round, RHE and NLT assembled a draft curriculum framework document, and this was also reviewed and refined through group discussion within the team. It was this draft that was presented in the community consultation.

### Community consultation phase

We did not include TGD community members in the Delphi phase of the study as we wanted to focus on the integrated and practical aspects of teaching TGD material at the UME level rather than on, say, the design and provision of medical services, which would reasonably have included TGD voices at an earlier stage. Nevertheless, reflecting our trauma-informed approach, we sought to include TGD perspectives and voices to ensure that our framework adequately covered the medical needs as identified by members of that community. Applying the principle of ‘nothing about us without us,’ we therefore sought their feedback on the draft curriculum framework. We were particularly interested in gauging levels of support and/or concern, in identifying any gaps or weaknesses in the material presented, and in grounding the material in the lived experiences of TGD Canadians. Initially we had planned to do this with in-person focus groups, but we switched to an online questionnaire in the interests of anonymity and confidentiality. To that end, a consultation questionnaire instrument was developed by RHE and NLT and iteratively reviewed and refined in discussion with the rest of the team.

A link to the questionnaire was circulated to local and national TGD groups and support networks via social media, national pride member lists, professional TGD organizations, community groups, word of mouth, and through a letter sent to TGD patients in a physician’s practice. Participation was entirely anonymous, and the instrument was left open for six weeks after invitations to participate were distributed.

Analysis of the responses was undertaken by RHE and NLT, separating comments on the framework from those about the framework. Comments on the framework were discussed by the team to develop consensus edits that reflected the suggestions and concerns raised. Comments about the framework were grouped and key issues and exemplifying quotes identified by RHE and NLT and added to the Discussion section.

### Final analysis and synthesis phase

Responses from the consultation were reviewed by RHE and NLT and changes made to the draft framework in terms of language used and in how items were presented and grouped. Broader community comments about the context for the framework are considered in the Discussion section. The resulting curriculum framework document was circulated within the team for several rounds of group writing, confirmation and comment before a final version was agreed upon.

#### Reflexivity:

The study team was led by a medical learner activist (NLT) and a PhD medical education researcher (RHE). The rest of the team was made up by three family medicine physician educators (TJJ, AH, RJ), five specialty physician educators (CTO, DP, HF, FM, JR), and a community organizer and liaison (JD). NLT was the study coordinator and drew on her extensive experience as a TGD activist, RHE brought her scientific expertise to the study, and the rest of the team brought their clinical and educational expertise as well as some research expertise.

#### Research ethics:

The study was approved by The University of Calgary Combined Health REB (REB18-1931) and The University of Toronto REB (#: 00037287).

#### Terminology:

We were unable to find an umbrella term that satisfied all the study participants. Although not widely used in the community, the term we used—transgender and gender diverse (TGD)—was the most widely accepted overall and it aligned best with language used in the research community.

## Results

**Fig. 2:**
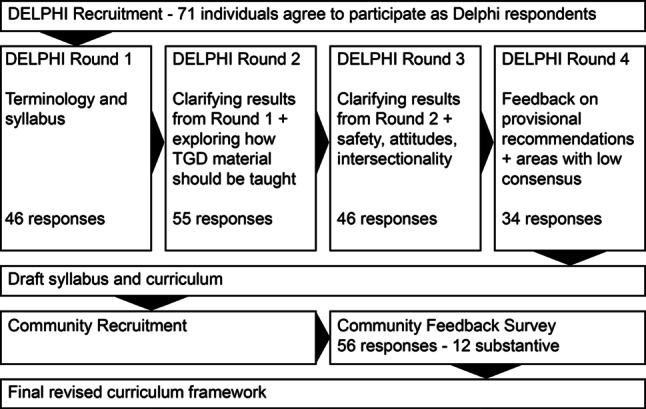
The flow of the study and the number of responses at each stage

The first round focused on 54 objectives (see Appendix 1 of the Electronic Supplementary Material) grouped by: Language and terminology; history; epidemiology; and social determinants of health; mental health; hormones; surgeries; routine primary care; and reproductive and sexual health. The second round presented the results from the first round for confirmation, and it also sampled opinions on the required outcomes from TGD training, and on who should teach, how much time should be allocated, which teaching and assessment modalities should be used, and the extent to which training should be mandatory. The third round focused on matters of safety, the learning environment, participation, and intersectionality (for instance with providing care for individuals with a Disorder of Sexual Differentiation). The fourth round presented the draft curriculum framework for confirmation and feedback. Changes were made to the draft in response to the ratings and comments from round four but as no notable disagreements emerged, we halted the Delphi process at this point (Fig. 2).

Although the ratings of round one items did not cluster around high and low aggregate scores, there was a notable drop in scores after #29. We therefore took the top 29 to continue to the second round along with six additional topics suggested by the reviewers. The other question-item rankings clustered around high- and low-ranked items, except for how much time should be dedicated to TGD teaching, which remained unresolved after two rounds. This was discussed within the team and it was dropped from the final framework.

A case for mandatory TGD training for future physicians has been made [32]. Our panelists agreed that the curriculum framework be a mandatory component of medical school education and that there be no basis for conscientious objection (Items 1.2.1 and 1.2.2). One participant stated:

I don’t think that it is reasonable to create a culture where it is allowable for students to be conscientious objectors to working with TGNC individuals. They may not need to personally agree, but I feel strongly that part of professionalism is being able to put aside your personal beliefs and treat patients who need treatment in a compassionate, empathetic matter.

Our panel, however, was unable to reach consensus on the number of hours required for teaching but, rather, agreed that the number of hours of TGD teaching time must be balanced with all other medical school curriculum demands.

Validation of the framework will rest in great part on how the medical education community responds to our findings. However, we did undertake the community consultation (56 responses) in part to contribute validation evidence and we present a selection of responses in that light. Generally, the feedback about the curriculum framework was highly positive and no major concerns or issues were raised:

I think that this curriculum goes a long way to address my concerns. I think that physicians coming out of this program will have a good basic understanding of how to treat and care for TGD patients.

It covers the basics that have been missed in many clinics, including the sensitivity needed.

I think this is vital and important work, since physicians who are knowledgeable in this field are so difficult to find, especially in more remote or socially conservative areas.

Thank you for reaching out to the community in creating this curriculum. Having community input like this is a big step in helping to heal the medical trauma many of us have faced.

However, there were issues raised that emphasized the need for this work and for change in healthcare as a whole. One participant commented: *“A lot of what we need is doctors to understand we actually exist”. *Others noted the need for an assured minimum standard of competence and knowledge:

I think the idea of a specialized curriculum for gender affirming care is long overdue. Having a set of standards for physician education will help with accountability and standardization of information regarding best practices to treat trans patients.

Some raised issues of safety and integrity based on their own experiences:

It would definitely be a relief to know that transphobia would be actively taught against. It would not prevent unpleasant/potentially dangerous interactions entirely, of course, but it would certainly make me feel a bit safer.

Providing a baseline understanding of trans medical needs and inclusive language shifts the burden of providing this education off the patient, allowing more time for comprehensive healthcare.

**Fig. 3:**
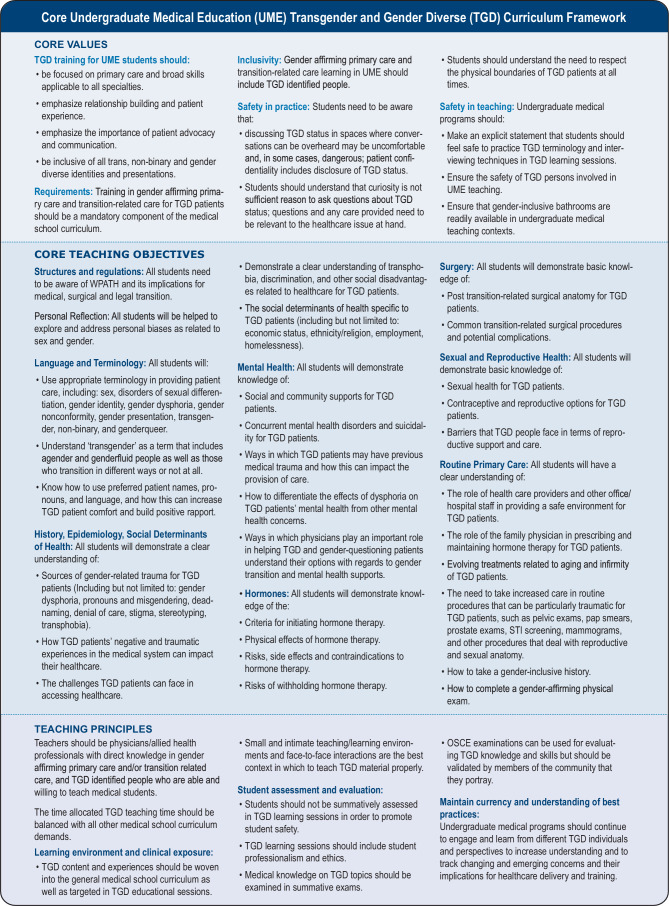
Core undergraduate medical education (UME) transgender and gender diverse (TGD) curriculum framework

See Fig. 3 for the overall curriculum framework. Note: a fuller version of the curriculum framework is provided in Appendix 2 of the Electronic Supplementary Material.

## Discussion

This study directly responds to the absence of clear curriculum guidelines for teaching TGD material raised by Nolan et al. [18] Our approach allowed us to bring together a variety of perspectives from across Canada including stakeholders from specialized TGD medical/surgical care, primary care, and undergraduate medical education. Involving TGD community members in the study team, as well as in the community consultation, ensured that our curriculum framework addressed health priorities identified by them.

Our framework emphasizes the inclusion of TGD community members in training activities. A recent study by Stroumsa et al. [29] noted that transphobic attitudes amongst medical students were negatively associated with objective measures of TGD knowledge independent of the number of curriculum hours spent on the material. Positive and frequent intergroup contact has also been correlated with more positive attitudes towards sexual minority groups [30], and we anticipated that direct contact would also have a positive impact on attitudes towards openly TGD-identified individuals. This did, however, raise the point about safety for TGD community members who offer to teach this content or share their personal stories with learners especially in smaller centers where anonymity may be harder to achieve. Panelists agreed that safety for those TGD persons involved in teaching is a top priority (Appendix 2, Item 1.5.2) but that each program would need to have an individualised approach to safety reflecting their local contexts and concerns.

The panel also agreed that students should feel safe practicing gender-affirming interviewing techniques and terminology. Our pilot study in 2017 identified that some learners were hesitant to participate in small group discussion fearing that they might inadvertently use language that could be considered insensitive or transphobic. If not addressed, this fear has the potential to reduce the effectiveness of any TGD curriculum. Appendix 2, Item 1.5.1 recommends that UME programs make an explicit statement that students should feel safe to practice TGD-related terminology and that honest learner error will be tolerated and corrected in a respectful, non-shame-based way. It was also recommended that students should not be assessed during TGD learning sessions so as to promote safety (Item 3.4.1), though there should be an evaluation of student professionalism and ethics (Item 3.4.2). This also supports the recommendation that physician or allied health instructors should have experience with the TGD population (Item 3.1.1) and that smaller and more intimate teaching environments are needed (Item 3.3.2).

In addition, we recognize that terminology is in constant flux which may be a source of frustration for learners who are looking for a concrete list of acceptable vs. unacceptable terms. Terminology was also a source of discussion within the study team leading us to change from “Transgender and Gender Non-Conforming” which was used in the first Delphi round to “Transgender and Gender Diverse” used in subsequent rounds. Although TGD was not a term used by the LGBTQ+ community, it is more commonly used in academic and medical literature. The term Gender Diverse may also be in flux given the use of Gender Expansive in some newer publications [31].

An important point that came forward was that TGD expertise is often incorrectly considered vitally relevant in the care of non-trans-related conditions. As one of our Delphi participants wryly observed:

The “trans cold” … is obviously different from the cis-gender cold and requires treatment in a gender clinic. It is important to educate clinicians about such nonsense.

However, thoughts on how this issue should be resolved varied significantly and consensus on its representation was hard to achieve. We elected therefore to use the language suggested by our community participants regarding curiosity and relevance (Item 1.4.2).

### Limitations

We note a number of limitations in this study. Firstly, recruitment of Delphi panelists proved challenging as many physicians and faculty (even those who organize TGD sessions) were reluctant to participate as they did not consider themselves as sufficiently knowledgeable. Nevertheless, and despite some attrition in the numbers participating, we had good representation from our different groups and from across Canada. We also acknowledge that, as our study was conducted in English, we were not as inclusive of French-speaking Canadians as we might have been. Our use of bimodality for consensus thresholds for ranked items, rather than predefined thresholds based on percentages, deviated from some interpretations of Delphi methods but it reflected the breadth of material covered and the intrinsic subjectivity of the judgments we presented our panel with. Given our already broad focus on TGD care we elected not to explore intersectionalities of TGD with other equity-seeking groups such as those identified around race and indigeneity, sexuality, culture, ethnicity, faith, and (dis)ability. Nevertheless, we recognize that these are important issues, and they should be explored in subsequent research. We also acknowledge the complex interplay between the needs and challenges of TGD and people with Disorders of Sexual Differentiation, which do not always align. Although the need for TGD training in medical education in developing countries has been established [33], our focus on a single country may limit the generalizability of our findings to other jurisdictions. We expand on this in the following section.

### Implementation

Defining a curriculum framework is an important step, but it will still need to be implemented. Medical school curricula are notoriously packed, and proposed additions and changes are often contested and compromised in the process of implementing them [34, 35]. We anticipate therefore that how this framework is implemented will depend to a great extent on local circumstances. We also note that TGD identities and expressions vary by culture (for instance two-spirit, hijra, and kathoey identities) which is also likely to have implementation implications [36].

While some customization and adaptation will likely be necessary, we also note that TGD rights and access to healthcare remains a politicized issue in many contexts around the world. Indeed, we note with some dismay that many states and jurisdictions around the world remain actively hostile towards their TGD populations, in many cases curtailing or inhibiting their access to care [37, 38]. Implementing an affirmative TGD curriculum in these contexts would likely be more challenging but no less important (and likely far more pressing) than in less systemically transphobic settings [12].

The competence and confidence of teachers to present this material is another concern one that has been noted by others [5, 18]. Based on our own experiences, this content is best taught by those who have a high degree of comfort/familiarity with providing care for TGD patients. Hopefully, as TGD care becomes more integrated across medical education and future physicians incorporate this care and knowledge into foundational primary care, expert preceptors may be less essential. Finally, although the panel did not reach consensus for the minimum number of hours that should be dedicated to TGD training, given that a third of US and Canadian schools in 2010 had no mandatory LGBTQ+ content whatsoever and those that did had relatively little on TGD care [5], we call for schools around the world to acknowledge their responsibility to prepare tomorrow’s doctors to provide competent and respectful healthcare services to and for TGD patients.

## Supplementary Information

Appendix 1: Delphi Instruments

Appendix 2: Core UME TGD Curriculum Framework
