# Supplementary material for: An undergraduate medical curriculum framework for providing care to transgender and gender diverse patients: A modified Delphi study
Source: Perspect Med Educ. 2021 Nov 18;11(1):36–44. doi: 10.1007/s40037-021-00692-7 (PMC8600495; doi:10.1007/s40037-021-00692-7)
Supplement: Supplementary file 1 — Appendix 1: Delphi Instruments [file 40037_2021_692_MOESM1_ESM.docx]

**Appendix 1: Delphi Instruments**

===

**Round 1**

This questionnaire is the first in the Delphi series and covers what should be taught at the undergraduate medical education (UME) level. Please respond as fully as you are able. Your answers will be collated, themed and used to determine future questionnaires with the purpose of determining an appropriately detailed syllabus for teaching medical students gender affirming primary care and transition related care for the transgender and gender diverse population.

*Language & Terminology*

Please rate how important it is to cover the following topics in teaching Gender-Affirming Primary Care and Transition-related care to undergraduate medical students: [options: Not at all important - Low importance - Moderately important – Important – Essential]

- Terminology and basic concepts – ie. sex, gender identity, gender dysphoria, gender nonconformity, gender presentation, transgender, non-binary, gender queer

Are there any other language and terminology topics that should be included?

*History, Epidemiology, Social Determinants of Health*

Please rate how important it is to cover the following topics in teaching Gender-Affirming Primary Care and Transition-related care to undergraduate medical students: [options: Not at all important - Low importance - Moderately important – Important – Essential]

- History of transgender and gender diverse rights in Canada
- Epidemiologic Considerations
- Gender diversity from a social and cultural perspective
- Name and gender marker change
- Social determinants of health specific to transgender and gender diverse patients – ie economic status, ethnicity/religion, employment, homelessness
- Issues with access to care
- Transphobia, discrimination, and other social disadvantages

Are there any other History, Epidemiology, Social Determinants of Health topics that should be included?

*Mental Health*

Please rate how important it is to cover the following topics in teaching Gender-Affirming Primary Care and Transition-related care to undergraduate medical students: [options: Not at all important - Low importance - Moderately important – Important – Essential]

- Criteria for diagnosing gender dysphoria in adults
- Criteria for diagnosing gender dysphoria in children and adolescents
- Sources of gender-related trauma - gender dysphoria, pronouns and misgendering, deadnaming, denial of care, stigma, stereotyping, transphobia
- Gender expression and role change
- Psychotherapy including counselling and support for changes in gender role
- Family therapy or support for family members
- Social support
- Concurrent mental health disorders
- Risks of withholding medical treatments
- Addictions amongst the gender diverse population

Are there any other Mental Health topics that should be included?

*Hormones*

Please rate how important it is to cover the following topics in teaching Gender-Affirming Primary Care and Transition-related care to undergraduate medical students: [options: Not at all important - Low importance - Moderately important – Important – Essential]

- Criteria for initiating hormone therapy
- GnRH Analogs
- Regimens for feminizing hormone therapy
- Regimens for masculinizing hormone therapy
- Physical effects
- Timeline to onset of effect and expected maximum effect
- Risks, side effects and contraindications to hormone therapy
- Monitoring of patients while on hormone therapy
- Reference ranges for blood work monitoring hormone levels

Are there any other Hormones topics that should be included?

*Surgeries*

Please rate how important it is to cover the following topics in teaching Gender-Affirming Primary Care and Transition-related care to undergraduate medical students: [options: Not at all important - Low importance - Moderately important – Important – Essential]

- Criteria for surgery
- Referral process for surgery (local context)
- Breast/Chest Surgical Options
- Breast/Chest Surgical Techniques and complications
- Genital surgical options (feminizing)
- Genital surgical techniques and complications (feminizing)
- Genital surgical options (masculinizing)
- Genital surgical techniques and complications (masculinizing)
- Other surgeries (ie. Rhinoplasty, thyroid chondroplasty)
- Voice feminization surgery
- Postoperative care

Are there any other Surgeries topics that should be included?

*Routine Primary Care*

Please rate how important it is to cover the following topics in teaching Gender-Affirming Primary Care and Transition-related care to undergraduate medical students: [options: Not at all important - Low importance - Moderately important – Important – Essential]

- Approaches to taking a gender-inclusive history
- Gender Affirming Physical Exam
- Gender expression as part of normal childhood development
- Non-surgical options for voice feminization
- Cancer screening (ie. Prostate exam, Mammography, Pap Smear)
- Management of pelvic pain, pain with orgasm and vaginal atrophy while on Testosterone
- Management of breakthrough/abnormal bleeding on Testosterone
- Trans Broken-Arm Syndrome
- Health complications as a result of hormone therapy
- Role of providers and staff in providing a safe environment for transgender and gender diverse patients

Are there any other Routine Primary Care topics that should be included?

*Reproductive and Sexual Health*

Please rate how important it is to cover the following topics in teaching Gender-Affirming Primary Care and Transition-related care to undergraduate medical students: [options: Not at all important - Low importance - Moderately important – Important – Essential]

- Sexual assault in the transgender and gender diverse population
- Reproductive options for transwomen (ie. Sperm preservation, surrogacy)
- Reproductive options for transmen (ie. Pregnancy, surrogacy, oocyte or embryo freezing)
- Birth Control
- Lactation and pregnancy in transmen
- Sexual health

Are there any other Reproductive and Sexual Health topics that should be included?

*General comments*

Do you have any other comments and observations regarding what we should be teaching undergraduate medical students about transgender and gender diverse health?

===

**Round 2**

Based on round 1 of the study, the consensus was that the following topics were most important in teaching UME students about TGD care. Please rate the priority of each topic in teaching undergraduate medical students: [Options: Low priority - do not include Medium priority, may include High priority, definitely include]

- Approaches to taking a gender-inclusive history
- Terminology and basic concepts – ie. sex, gender identity, gender dysphoria, gender nonconformity, gender presentation, transgender, non-binary, gender queer
- Role of providers and staff in providing a safe environment for transgender and gender diverse patients
- Issues with transgender and gender diverse access to care
- Gender affirming physical exam
- Gender expression as part of normal childhood development
- Transphobia, discrimination, and other social disadvantages
- Risks of withholding medical treatments for transgender and gender diverse patients
- Social determinants of health specific to transgender and gender diverse patients – ie economic status, ethnicity/religion, employment, homelessness
- Sexual health for transgender and gender diverse patients
- Sources of gender-related trauma - gender dysphoria, pronouns and misgendering, deadnaming, denial of care, stigma, stereotyping, transphobia
- Criteria for initiating hormone therapy for transgender and gender diverse patients
- Cancer screening for transgender and gender diverse patients (ie. prostate exam, mammography, pap smear)
- Concurrent mental health disorders for transgender and gender diverse patients
- Sexual assault in the transgender and gender diverse population
- Criteria for diagnosing gender dysphoria in adults
- Health complications as a result of hormone therapy for transgender and gender diverse patients
- Birth control for transgender and gender diverse patients
- Criteria for diagnosing gender dysphoria in children and adolescents
- Risks, side effects and contraindications to hormone therapy for transgender and gender diverse patients
- Monitoring of transgender and gender diverse patients while on hormone therapy
- Gender diversity from a social and cultural perspective
- Forms and aspects of gender expression and role change
- Name and gender marker changes, documentation issues
- Psychotherapy including counselling and support for changes in gender role
- Criteria for surgery for transgender and gender diverse patients
- Referral process for surgery (local context)
- Physical effects of hormones for transgender and gender diverse patients
- Social supports for transgender and gender diverse patients

Based on your comments and suggestions in round 1, a number of additional topics were noted. Please rate the priority of each topic in teaching undergraduate medical students: [options: Low priority - do not include Medium priority, may include High priority, definitely include]

- How negative and traumatic experiences in the medical system for transgender and gender diverse patients impacts quality of and access to care
- Specific considerations for youth transgender and gender diverse patients, including age of consent and legally permissible interventions
- Suicide in the transgender and gender diverse patient population
- The role of the family physician in prescribing and maintaining hormone therapy for for transgender and gender diverse patients
- Basic overview of post-surgical anatomy for for transgender and gender diverse patients (ie. vaginoplasty, metoidioplasty, phalloplasty, chest reconstruction)
- Financial cost of for transgender and gender diverse related surgeries in Canada

Are there any other topics missing?

Do you have any suggested changes to the wording on any of these topics?

*Outcomes*

How important are the following graduating outcomes of teaching gender affirming and transition related care to undergraduate medical students? [Options: Not at all important A little important Quite important - Very important Extremely important]

- Demonstrate competence and confidence in using terminology as it relates to sex, gender, anatomy and sexual health.
- Demonstrate ability to describe primary care assessment and screening commensurate with the physical attributes of the patient
- Understand medical therapies and their side effects
- Understand gender affirming surgical procedures and their outcomes
- Advocate for TGD patients and their healthcare
- Understand potential provider/system barriers to TGD patients’ accessing health care.
- Build rapport and relationships with TGD patients
- Identify and address personal biases as related to sex and gender
- Be aware of general issues regarding TGD health
- Demonstrate a continued commitment to learning about TGD community
- Develop skills in gender affirming approaches to sexual health and history taking as it relates to gender
- Keep up to date on the science of TGD healthcare
- Keep up to date on social and legal issues regarding TGD individuals
- Teach others about TGD healthcare
- Conduct research and other scholarship regarding TGD healthcare

Are there any other outcomes for UME students that should be added to the list?

*Curriculum*

To what extent should the following people be involved in teaching gender affirming and transition related care to undergraduate medical students? [options: Low priority – do not involve Medium priority, may involve High priority, definitely involve]

- Preceptors with substantial experience in providing transgender and gender diverse healthcare
- Preceptors with some experience in providing transgender and gender diverse healthcare
- Preceptors with no experience in providing transgender and gender diverse healthcare
- Allied health professionals with substantial experience in providing transgender and gender diverse healthcare
- Allied health professionals with some experience in providing transgender and gender diverse healthcare
- Allied health professionals with no experience in providing transgender and gender diverse healthcare
- Transgender and gender diverse community members currently in transition or receiving gender confirming care
- Transgender and gender diverse community members who have transitioned and/or completed gender confirming care
- Family members of transgender and gender diverse persons

Are there any other persons that should be involved in teaching gender affirming and transition related care to undergraduate medical students?

How much time during the preclinical phase should be given to teaching gender affirming and transition related care to medical students?

- <1 hour
- 1-3 hours
- 3-6 hours
- 6-12 hours
- 12-20 hours
- More than 20 hours

How much time during clerkship should be given to teaching gender affirming and transition related care to medical students?

- <1 hour
- 1-3 hours
- 3-6 hours
- 6-12 hours
- 12-20 hours
- More than 20 hours

Do you have any comments on the time allocated to teaching gender affirming and transition related care to medical students?

How should gender affirming and transition related care be taught to undergraduate medical students? [options: Do not use Use a little Use a lot]

- Dedicated large group lectures/presentations/podcasted content
- Dedicated large group patient presentation or panel
- Interactive online learning modules
- Dedicated small group learning
- Dedicated practical skills sessions
- Dedicated standardized patient interviews
- Part of lectures on other topics
- Part of small group learning on other topics
- Part of practical skills sessions on other topics
- Optional sessions (invited speakers, Student Interest Groups)
- Clinical experience (shadowing)
- Clinical experience (service learning)

What other approaches to teaching gender affirming and transition related care to undergraduate medical students should we use?

- To what extent should gender affirming and transition related care be a mandatory part of the undergraduate medical curriculum?
- All aspects mandatory for all students as part of core curriculum
- Mandatory core curriculum plus optional sessions
- Optional sessions within the curriculum
- Optional sessions outside the curriculum

Do you have any comments on the extent to which gender affirming and transition related care should be a mandatory part of the undergraduate medical curriculum?

How should gender affirming and transition related care be assessed for undergraduate medical students? [options: Do not use Use a little Use a lot]

Written exams

Practical exams (OSCEs)

Clinical observations (bedside)

Professionalism and ethics

Do you have any comments on how gender affirming and transition related care should be assessed in the undergraduate medical curriculum?

Do you have any other comments and observations regarding TGD health in the context of the undergraduate medical curriculum?

===

**Round 3**

Thank you for participating in rounds 1 and 2 of the study. This final round focuses on the social and affective dimensions of TGNC teaching for UME students.

*Safety*

Learner safety - How likely are the following to occur in teaching TGNC topics in UME? [options: Very likely - Moderately likely - Neither likely nor unlikely - Moderately unlikely - Very unlikely - No opinion/unsure]

- Learner embarrassment or discomfort with the topic
- Learner embarrassment or discomfort with interacting with TGNC individuals
- Learner discomfort due to fear of saying something insensitive accidentally or using the wrong pronoun unintentionally or otherwise being mistakenly offensive
- Discomfort or distress of learners who are themselves TGNC or who have friends or relatives who are TGNC
- Hostile, disrespectful, or transphobic learner behaviours
- Lack of interest or engagement from learners

Teacher safety - How likely are the following to occur in teaching TGNC topics in UME [options: Very likely - Moderately likely - Neither likely nor unlikely - Moderately unlikely - Very unlikely - No opinion/unsure]

- Teacher embarrassment or discomfort with the topic
- Teacher embarrassment or discomfort with interacting with TGNC individuals
- Teacher fear of saying something insensitive accidentally or using the wrong pronoun unintentionally or being mistakenly offensive
- Hostile, disrespectful, or transphobic teacher behaviour
- Lack of interest or engagement from teachers

TGNC community member safety - How likely are the following to occur in teaching TGNC topics in UME? [options: Very likely - Moderately likely - Neither likely nor unlikely - Moderately unlikely - Very unlikely - No opinion/unsure]

- TGNC community member embarrassment or discomfort
- Hostile or disrespectful TGNC community member behaviour
- Unsafe environment for TGNC community member

What other safety concerns associated with TGNC teaching at the UME level are there? If you have had firsthand experience of safety matters in TGNC teaching please note them here.

*Environment*

Please rate the importance of the following steps in establishing a safe environment for teaching TGNC topics in UME: [options: Critical importance High importance Moderate importance Low importance No importance No opinion/unsure]

- Briefing and preparation for learners – what to expect, rules of engagement
- Briefing and preparation for teachers – what to expect, rules of engagement
- Briefing and preparation for community members – what to expect, rules of engagement
- Gender neutral bathroom availability
- Statement from UME that students should feel safe to practice TGD language and interviewing techniques and that feedback will be free of judgement or shaming.
- Allow students who don’t want to be there to opt out of the session
- Allow physician instructors to opt out of teaching a session
- Careful selection of instructors for the sessions
- Pair TGNC identified instructor with physician instructor
- Debriefing TGNC teaching sessions

What other steps should be taken in establishing a safe environment for teaching TGNC topics in UME?

*Participation*

Please rate the acceptability of the following potential reasons a medical student may feel reluctant to participate in a TGNC teaching session: [options: Completely acceptable Moderately acceptable Neither acceptable or unacceptable Moderately unacceptable Completely unacceptable No opinion/unsure]

- Declaration of personal discomfort with TGNC persons or issues
- Religious or cultural concerns with TGNC individuals or care
- Declaration of personal belief that transgender identities are invalid
- Declaration of personal belief that gender is strictly binary
- Student who is TGNC and has concerns regarding their own safety or experience
- Student states that they will never practice medicine with gender diverse or transgender people

Are there any acceptable reasons for students to opt out of TGNC teaching?

If a medical student is identified by UME as unwilling to engage with TGNC care, to what extent should the UME be responsible for challenging that belief?

- Exclusive responsibility
- Major responsibility
- Minor responsibility
- No responsibility
- No opinion/unsure

Do you have any comments about medical student unwillingness to engage with TGNC care?

If a medical teacher is identified by UME as unwilling to engage with TGNC care, to what extent should the UME be responsible for challenging that belief?

- Exclusive responsibility
- Major responsibility
- Minor responsibility
- No responsibility
- No opinion/unsure

Do you have any comments about medical teacher unwillingness to engage with TGNC care?

*Intersectional issues*

How important is it that gender binaries be challenged in medical education?

- Critical importance
- High importance
- Moderate importance
- Low importance
- No importance
- No opinion/unsure

How important is it to teach TGNC care in terms of its intersections with the following topics: [options: Critical importance High importance Moderate importance Low importance No importance No opinion/unsure]

- Social determinants of health
- Race and ethnicity
- Culture
- Socioeconomic status
- How to advocate for TGNC individuals in general
- How to combat transphobia and discrimination against TGNC individuals in general

What other intersectional topics should be a part of TGNC teaching in UME?

How important is it that intersex topics should also be taught in UME?

- Critical importance
- High importance
- Moderate importance
- Low importance
- No importance
- No opinion/unsure

What relative priority should be given to TGNC and intersex teaching in UME?

- Teaching intersex topics is much more important than teaching TGNC topics in UME
- Teaching intersex topics is moderately more important than teaching TGNC topics in UME
- Teaching intersex topics is of equal importance to teaching TGNC topics in UME
- Teaching TGNC topics is moderately more important than teaching intersex topics in UME
- Teaching TGNC topics is much more important than teaching intersex topics in UME

To what extent should TGNC and intersex topics be taught together?

- TGNC and intersex topics should be fully taught together
- TGNC and intersex topics should be partly taught together
- TGNC and intersex topics should be taught completely separately
- Do you have any comments about TGNC and intersex teaching in UME?

*Closing comments*

Do you have any final comments on teaching TGNC care at the UME level?

===

**Round 4**

Thank you for your continued contribution to this process. This is the fourth and final consensus building round and will be your last opportunity to provide input. Following this round, our recommendations will be evaluated by a panel of TGD identified people and families/loved ones of TGD identified people in focus groups. In this round we are seeking your input on our provisional recommendations as well as your comments on the areas where we have not had consensus. We have structured our questions around three areas: core syllabus, core values, and core curriculum

*Terminology*

In discussions within the team we have shifted terminology for this round from TGNC (transgender and gender non-conforming) to TGD (transgender and gender diverse).

To what extent do you agree changing the terminology from TGNC (transgender and gender non-conforming) to TGD (transgender and gender diverse): [options: Strongly disagree - Disagree – Neutral - Agree - Strongly Agree

Your comments on the change in terminology:

*Core Syllabus*

In rounds 1 and 2 we identified the following as the core mandatory syllabus for TGD health training in Undergraduate Medical Education:

- Personal Reflection
- The ability to identify and address personal biases as related to sex and gender
- Language & Terminology
- The ability to use appropriate terminology and basic concepts in the context of providing patient care (Including but not limited to: sex, disorders of sexual differentiation, gender identity, gender dysphoria, gender nonconformity, gender presentation, transgender, non-binary, genderqueer)
- History, Epidemiology, Social Determinants of Health
- Sources of gender-related trauma (Including but not limited to: gender dysphoria, pronouns and misgendering, deadnaming, denial of care, stigma, stereotyping, transphobia)
- A clear understanding of how negative and traumatic experiences in the medical system impacts health care
- A clear understanding of issues with transgender and gender diverse access to care
- A clear understanding of transphobia, discrimination, and other social disadvantages related to healthcare for TGD individuals
- A clear understanding of the social determinants of health specific to TGD individuals (including but not limited to: economic status, ethnicity/religion, employment, homelessness)
- Mental Health
- Social and community supports
- Concurrent mental health disorders and suicidality
- Hormones
- Risks, side effects and contraindications to hormone therapy
- Physical effects of hormone therapy
- Criteria for initiating hormone therapy
- Surgery
- Basic overview of post-surgical anatomy for TGD persons (Including but not limited to: Vaginoplasty, Metoidioplasty, Phalloplasty, Chest reconstruction)
- Routine Primary Care
- Role of providers and staff in providing a safe environment
- The role of the family physician in prescribing and maintaining hormone therapy
- Approaches to taking a gender-inclusive history
- Gender affirming physical exam
- Risks of withholding hormone therapy
- Sexual health
- Cancer screening (Including but not limited to: prostate exam, mammography, pap smear)
- Contraceptive and reproductive options

To what extent do you agree with this syllabus for TGD health training at the UME level: [options: Strongly disagree – Disagree – Neutral – Agree - Strongly Agree]

Your comments on the syllabus – note: if you do have concerns (neutral/disagree ratings), please ensure that your concerns are clearly articulated here:

*Core Values*

In rounds 1 and 2 we identified the following core values for TGD health training in Undergraduate Medical Education:

- Generalist scope
  - TGD training should be focused on primary care and broad skills that are applicable to all specialties
  - TGD training should emphasize CanMEDs Roles: Advocate and Communicator
  - TGD training should emphasize relationship building and patient experience
- Mandatory
  - All students enrolled in undergraduate medical programs across Canada should receive training in caring for TGD patients.
  - TGD training should be a mandatory component of medical school curriculum.
  - There is no acceptable option for students’ conscientious objection to participating in TGD training unless a student who identifies as TGD has concerns about their personal wellness or safety.
- Safety
  - UME should make an explicit statement that students should feel safe to practice TGD terminology and interviewing techniques in TGD learning sessions
  - Gender neutral bathrooms should be available
- Inclusivity
  - TGD learning activities in UME should include TGD identified people

At the UME level, to what extent do you agree with these core values for TGD health training at the UME level? [options: Strongly disagree – Disagree – Neutral – Agree - Strongly Agree]

Your comments on the core values – note: if you do have concerns (neutral/disagree ratings), please ensure that your concerns are clearly articulated here:

*Core Curriculum*

Please rate the following statements on faculty involved in TGD health training at the UME level: [options: Strongly disagree – Disagree – Neutral – Agree - Strongly Agree]

- TGD content should be taught by physicians with specialized knowledge in TGD health care
- TGD content should be taught by allied health care professionals with specialized knowledge in TGD health care
- TGD content should be taught by people who identify as TGD and are able and willing to teach medical students

Comments on these statements – note: if you do have concerns (neutral/disagree ratings), please ensure that your concerns are clearly articulated here:

Please rate the following statements on the time allocated to TGD health training: [options: Strongly disagree – Disagree – Neutral – Agree - Strongly Agree]

- Number of hours of recommended teaching time must be balanced with all other UME curriculum demands
- There should be a minimum acceptable number of hours allocated to TGD training
- TGD content and experiences should be woven into general UME curriculum
- TGD content and experiences should be delivered in targeted TGD sessions

Comments on these statements – note: if you do have concerns (neutral/disagree ratings), please ensure that your concerns are clearly articulated here:

Please rate the following statements on instructional approaches in TGD health training at the UME level: [options: Strongly disagree – Disagree – Neutral – Agree - Strongly Agree]

- Smaller interactive group teaching/learning environments and face-to-face interactions are needed to teach TGD material properly.
- Clinical exposure to TGD patients in clerkship should be mandatory.
- TGD patients should be involved in TGD training but only if their safety is a top priority for all concerned.

Comments on these statements – note: if you do have concerns (neutral/disagree ratings), please ensure that your concerns are clearly articulated here:

Please rate the following statements on evaluation of UG students in TGD health training at the UME level: [options: Strongly disagree – Disagree – Neutral – Agree - Strongly Agree]

- TGD learning sessions should be formative in order to promote learner safety
- TGD learning sessions should include a component on evaluating student professionalism and ethics
- Medical knowledge on TGD topics should be examined in paper-based examinations
- OSCE examinations should be used for evaluating TGD knowledge and skills
- Assessments and evaluations of TGD knowledge and skills should be validated by members of the TGD community

Comments on these statements – note: if you do have concerns (neutral/disagree ratings), please ensure that your concerns are clearly articulated here:

*Final comments*

Do you have any final comments for the study as a whole?
