# Supplementary material for: An undergraduate medical curriculum framework for providing care to transgender and gender diverse patients: A modified Delphi study
Source: Perspect Med Educ. 2021 Nov 18;11(1):36–44. doi: 10.1007/s40037-021-00692-7 (PMC8600495; doi:10.1007/s40037-021-00692-7)
Supplement: Supplementary file 2 — Appendix 2: Core UME TGD Curriculum Framework [file 40037_2021_692_MOESM2_ESM.docx]

**Appendix 2: Core UME TGD Curriculum Framework**

1. **Core Values**
   1. *Scope:* TGD training for undergraduate medical students should:
      1. be focused on primary care and broad skills that are applicable to all specialties.
      2. emphasize relationship building and patient experience.
      3. emphasize the importance of patient advocacy and communication.
      4. be inclusive of all trans, non-binary and gender diverse identities and presentations.
   2. *Requirements:*
      1. All students enrolled in undergraduate medical programs should receive training in caring for TGD patients.
      2. Training in gender affirming primary care and transition-related care for TGD patients should be a mandatory component of the medical school curriculum.
   3. *Inclusivity:*
      1. Gender affirming primary care and transition-related care learning activities in medical education should include TGD identified people.
   4. *Safety in practice:*
      1. Students need to be aware that discussing TGD status in spaces where conversations can be overheard may be uncomfortable and, in some cases, dangerous; patient confidentiality includes disclosure of TGD status.
      2. Students should understand that curiosity is not sufficient reason to ask questions about TGD status; questions and any care provided need to be relevant to the healthcare issue at hand.
      3. Students should understand the need to respect the physical boundaries of TGD patients at all times.
   5. *Safety in teaching:* Undergraduate medical programs should:
      1. Make an explicit statement that students should feel safe to practice TGD terminology and interviewing techniques in TGD learning sessions.
      2. Ensure the safety of TGD persons who are involved in undergraduate medical teaching.
      3. Ensure that gender-inclusive bathrooms are readily available in undergraduate medical teaching contexts.
2. **Core Teaching Objectives**

*2.1 Awareness of structures and regulations*

2.1.1 All students need to be aware of WPATH and its implications for medical, surgical and legal transition.

*2.2 Personal Reflection:* All students will:

2.2.1 Be helped to explore and address personal biases as related to sex and gender.

*2.3 Language & Terminology:* All students will:

- - 1. Use appropriate terminology and basic concepts in the context of providing patient care for TGD patients, including but not limited to: sex, disorders of sexual differentiation, gender identity, gender dysphoria, gender nonconformity, gender presentation, transgender, non-binary, and genderqueer.
    2. Understand that ‘transgender’ is an umbrella term used in the LGBTQ community that can include people who are agender and genderfluid as well as those who may transition in different ways or not at all.
    3. Know how to use preferred patient names, pronouns, and language, and understand how this can increase TGD patient comfort and build positive rapport.

*2.4 History, Epidemiology, Social Determinants of Health :* All students will demonstrate a clear understanding of:

2.4.1 Sources of gender-related trauma for TGD patients (Including but not limited to: gender dysphoria, pronouns and misgendering, deadnaming, denial of care, stigma, stereotyping, transphobia).

2.4.2 How TGD patients’ negative and traumatic experiences in the medical system can impact their healthcare.

2.4.3 The challenges TGD patients can face in accessing healthcare.

2.4.4 Demonstrate a clear understanding of transphobia, discrimination, and other social disadvantages related to healthcare for TGD patients.

2.4.5 The social determinants of health specific to TGD patients (including but not limited to: economic status, ethnicity/religion, employment, homelessness).

*2.5 Mental Health:* All students will demonstrate knowledge of:

2.5.1 Social and community supports for TGD patients.

2.5.2 Concurrent mental health disorders and suicidality for TGD patients.

2.5.3 Ways in which TGD patients may have previous medical trauma and the ways in which this can impact the provision of care.

2.5.4 How to differentiate the effects that dysphoria has on for TGD patients’ mental health from other mental health concerns.

2.5.5 Ways in which physicians play an important role in helping TGD and gender-questioning patients understand their options with regards to gender transition and mental health supports.

*2.6 Hormones:* All students will demonstrate knowledge of the:

2.6.1 Criteria for initiating hormone therapy for TGD patients.

2.6.2 Physical effects of hormone therapy for TGD patients.

2.6.3 Risks, side effects and contraindications to hormone therapy for TGD patients.

2.6.4 Risks of withholding hormone therapy for TGD patients.

*2.7 Surgery:* All students will demonstrate basic knowledge of:

2.7.1 Post transition-related surgical anatomy for TGD patients (including but not limited to: vaginoplasty, metoidioplasty, phalloplasty, chest reconstruction, facial feminization surgery)

2.7.2 Common transition-related surgical procedures and potential complications for TGD patients (such as pain, infection, bleeding, hypergranulation tissue, internal hair, etc.)

*2.8 Sexual and Reproductive Health:* All students will demonstrate basic knowledge of:

2.8.1 Sexual health for TGD patients.

2.8.2 contraceptive and reproductive options for TGD patients.

2.8.3 barriers that TGD people face in terms of reproductive support and care.

*2.9 Routine Primary Care:* All students will have a clear understanding of:

2.9.1 The role of health care providers and other office/hospital staff in providing a safe environment for TGD patients.

- - 1. The role of the family physician in prescribing and maintaining hormone therapy for TGD patients.
    2. Evolving treatments related to aging and infirmity of TGD patients.
    3. The need to take increased care in routine procedures that can be particularly traumatic for TGD patients, such as pelvic exams, pap smears, prostate exams, STI screening, mammograms, and other procedures that deal with reproductive and sexual anatomy, and how to handle potential adverse reactions to such procedures.

2.9.4 How to take a gender-inclusive history.

2.9.5 How to complete a gender-affirming physical exam.

1. **Teaching Principles**
   1. Teachers should be physicians or allied health professionals with direct knowledge in gender affirming primary care and/or transition related care, and TGD identified people who are able and willing to teach medical students.

*3.2* The time allocated TGD teaching time should be balanced with all other medical school curriculum demands.

*3.3 Learning environment and clinical exposure:*

3.3.1 TGD content and experiences should be woven into the general medical school curriculum as well as targeted in TGD educational sessions.

3.3.2 Small and intimate teaching/learning environments and face-to-face interactions are the best context in which to teach TGD material properly.

- 1. *Student assessment and evaluation:*
     1. Students should not be summatively assessed in TGD learning sessions in order to promote student safety.
     2. TGD learning sessions should include student professionalism and ethics.
     3. Medical knowledge on TGD topics should be examined in summative exams.
     4. OSCE examinations can be used for evaluating TGD knowledge and skills but should be validated by members of the community that they portray.
  2. *Maintain currency and understanding of best practices:* Undergraduate medical programs should continue to engage and learn from different TGD individuals and perspectives to increase understanding and to track changing and emerging concerns and their implications for healthcare delivery and training.
